# Validation of the reshaped shared epitope HLA-DRB1 classification in rheumatoid arthritis

**DOI:** 10.1186/ar1949

**Published:** 2006-04-28

**Authors:** Laëtitia Michou, Pascal Croiseau, Elisabeth Petit-Teixeira, Sophie Tezenas du Montcel, Isabelle Lemaire, Céline Pierlot, José Osorio, Wafa Frigui, Sandra Lasbleiz, Patrick Quillet, Thomas Bardin, Bernard Prum, Françoise Clerget-Darpoux, François Cornélis

**Affiliations:** 1 GenHotel-EA 3886, University Evry-ParisVII Medical School, Member of the Autocure European Consortium, Evry-Genopole, France; 2 INSERM U 535, University Paris XI, Villejuif, France; 3 Service de Biostatistiques et d'Information Médicale, CHU Pitié-Salpétrière, Assistance Publique-Hôpitaux de Paris, Paris, France; 4 Laboratoire de Biologie, Centre Hospitalier Sud Francilien, Corbeil-Essonne, France; 5 Unité de Génétique Clinique Adulte, Hôpital Lariboisière, Assistance Publique-Hôpitaux de Paris, Paris, France; 6 Laboratoire Statistique et Génome, Evry-Genopole, France; 7 Consultation de Génétique Adulte, Centre Hospitalier Sud Francilien, Evry-Corbeil, France

## Abstract

Recently, we proposed a classification of HLA-DRB1 alleles that reshapes the shared epitope hypothesis in rheumatoid arthritis (RA); according to this model, RA is associated with the RAA shared epitope sequence (72–74 positions) and the association is modulated by the amino acids at positions 70 and 71, resulting in six genotypes with different RA risks. This was the first model to take into account the association between the HLA-DRB1 gene and RA, and linkage data for that gene. In the present study we tested this classification for validity in an independent sample. A new sample of the same size and population (100 RA French Caucasian families) was genotyped for the HLA-DRB1 gene. The alleles were grouped as proposed in the new classification: S_1 _alleles for the sequences A-RAA or E-RAA; S_2 _for Q or D-K-RAA; S_3D _for D-R-RAA; S_3P _for Q or R-R-RAA; and X alleles for no RAA sequence. Transmission of the alleles was investigated. Genotype odds ratio (OR) calculations were performed through conditional logistic regression, and we tested the homogeneity of these ORs with those of the 100 first trio families (one case and both parents) previously reported. As previously observed, the S_2 _and S_3P _alleles were significantly over-transmitted and the S_1_, S_3D _and X alleles were under-transmitted. The latter were grouped as L alleles, resulting in the same three-allele classification. The risk hierarchy of the six derived genotypes was the same: (by decreasing OR and with L/L being the reference genotype) S_2_/S_3P_, S_2_/S_2_, S_3P_/S_3P_, S_2_/L and S_3P_/L. The homogeneity test between the ORs of the initial and the replication samples revealed no significant differences. The new classification was therefore considered validated, and both samples were pooled to provide improved estimates of RA risk genotypes from the highest (S_2_/S_3P _[OR 22.2, 95% confidence interval 9.9–49.7]) to the lowest (S_3P_/L [OR 4.4, 95% confidence interval 2.3–8.4]).

## Introduction

The pathogenesis of rheumatoid arthritis (RA) is multifactorial, involving both genetic and environmental factors. Although associations between some HLA-DRB1 alleles and RA were reported nearly three decades ago, the biological mechanism underlying this association remains unknown. The presence of the RAA sequence at positions 72–74 of the HLA-DR β-chain molecule for all HLA-DRB1 alleles known to be associated with RA led to the shared epitope (SE) hypothesis [1]. This hypothesis received support from numerous case-control association studies in both Caucasian and non-Caucasian populations. However, studies testing the SE hypothesis have rejected this simple model, which stipulates that each SE allele confers the same risk [2-5].

Recently, Tezenas du Montcel and coworkers proposed a model of the SE component in RA [6]. Those investigators reconsidered the SE hypothesis and generated a new classification of HLA-DRB1 alleles, based on their investigation using the MASC (Marker Association Segregation Chi Square) method [7], which was conducted in 100 trio families (one case and both parents) and 132 index cases from affected sibling pair families, all from the French Caucasian population. They proposed that the risk for developing RA depends on whether the RAA sequence occupies positions 72–74 and, if this is the case, on the amino acids at positions 71 and 70. For those RAA alleles, lysine (K) at position 71 conferred the highest risk, arginine (R) an intermediate risk, and alanine (A) or glutamic acid (E) the lowest risk. Glutamine (Q) or arginine (R) at position 70 conferred greater risk than did aspartic acid (D). This resulted initially in five allele groups, which were simplified to three allele groups defining six genotypes with different RA risks. This study was the first to model the HLA component in RA taking into account both association and linkage data, resulting in a reshaped SE hypothesis.

Here, we tested this classification for validity by replication in a new, independent sample of 100 French Caucasian trio families, evaluating the risk hierarchy of the proposed classification for homogeneity with that of the initial sample.

## Materials and methods

### Study design and study population

An association study using conditional logistic regression was performed to investigate the hierarchy of risks associated with HLA-DRB1 genotypes in an independent sample of trio families. The new independent sample (sample B), similar to that used to generate the new classification (sample A), included 100 trio families (one RA patient and both parents) of French Caucasian origin (criteria fulfilled for each of the four grandparents). DNA from all of the trio families included in samples A and B was collected between 1994 and 1998, as were initial clinical characteristics of the RA index patients. RA diagnosis met the 1987 American College of Rheumatology (formerly, the American Rheumatism Association) criteria [8]. All individuals provided written informed consent, and the study was approved by the Hospital Bicêtre ethics committee (Kremlin-Bicêtre, Assistance Publique-Hôpitaux de Paris).

Clinical characteristics were updated in 2001 and 2002 for sample A and in 2004 for sample B. Four RA index patients in sample A and two RA index patients in sample B died between the time of DNA collection and the present study. The updated clinical characteristics of sample B were similar to those of sample A (the initial sample): 90% of RA patients in sample B were female versus 87% in sample A; the mean (± standard error) age at RA onset was 31 ± 9 years versus 32 ± 10 years; the mean (± standard error) disease duration was 16 ± 8 years versus 18 ± 7 years; erosions were present in 79% versus 90%; 76% were positive for serum rheumatoid factor versus 81%; and nodules were present in 19% versus 31%. Rheumatoid factor was considered positive when there was at least one positive rheumatoid factor finding during the course of the disease, as determined using latex fixation, Waaler Rose assay, or laser nephelometry.

### HLA-DRB1 genotyping

Blood samples were collected for DNA extraction and genotyping. HLA-DRB1 typing was performed using the polymerase chain reaction-sequence specific primer (SSP) method using Dynal Classic SSP DR low resolution and the Dynal Classic high resolution SSP (Dynal Biotech, Lake Success, NY, USA) for subtyping of HLA-DRB1*01, *04, *11, *13 and *15 alleles. Sequencing of exon 2 of HLA-DRB1 was performed for all four HLA-DRB1*04 alleles, ambiguous with the Dynal Classic method. HLA-DRB1 allele frequencies of control genotypes (obtained by combining untransmitted parental alleles for each family) were similar between samples and were comparable to the allele frequencies reported for the French population in the 11th Histocompatibility Workshop [9].

### HLA-DRB1 allele classification

HLA-DRB1 alleles were divided in two groups according to the presence or absence of the RAA sequence at positions 72–74, defining S and X alleles (Table 1). The S alleles were then subdivided into three categories, according to amino acid at position 71, as follows: S_1 _when an alanine or a glutamic acid was present at position 71 (A-RAA or E-RAA sequences; A-RAA alleles were too infrequent not to be pooled, as described previously [6]); S_2 _when a lysine was present (K-RAA sequence); and S_3 _when an arginine was present at position 71 (R-RAA sequence). Then S_3 _alleles were subdivided according to amino acid at position 70: S_3D _alleles encoding the D-R-RAA sequence and S_3P _alleles encoding the Q or R-R-RAA sequence. Because the S_2 _alleles had either Q or D at position 70, they had – by this '70-71-72/74' nomenclature – the Q or D-K-RAA sequence.

### Statistical analysis

We first investigated transmission of the five alleles (S_1_, S_2_, S_3D_, S_3P _and X) using a χ^2 ^test with one degree of freedom for each allele. Alleles with significant over-transmission from heterozygous parents to RA patients (>50%) are linked to and associated with RA. Alleles with significant under-transmission (<50%) exhibit no RA association and could be pooled for further analysis.

Then, for each genotype 'I', the odds ratio 'ORi' relative to a reference genotype and 95% confidence interval (CI) were calculated by conditional logistic regression. In this analysis, the genotypes observed for the RA patients were conditioned to the parents' genotypes [10,11]. The RA patient genotypes were compared using a likelihood ratio test with the pseudo-controls (i.e. the three other genotypes that could be formed by parental gametes). Given reference genotype with baseline risk termed β_0_, each OR β_i _(i = 1 ... *n*) was estimated by the maximization of the log likelihood (L):

ln(L) = β_0 _+ β_1_X_1 _+ β_2_X_2 _+ ... + β_*n*_X_*n*_

Where Xi is an indicator taking value 1 for genotype 'i' and 0 for the other genotypes, and β_i _= log ORi, with β_0 _being the baseline risk for reference genotype. Likelihood computations and estimation were performed using the program developed by Clayton [12]. All the results were produced using STATA software (David Clayton, Cambridge, UK).

In case of replication of the genotype risk hierarchy, a homogeneity test on genotypic ORs was performed between the two trio family samples. In this test, we considered that, if homogeneity was present, then Q = -2(ln(maxL_AB_) - (ln(maxL_A_) + (ln(maxL_B_)))) would follow a χ^2 ^distribution with *n *degrees of freedom (*n *being the number of β_i_s estimated). L_A_, L_B _and L_AB _were the maximum likelihood over β_i _in sample A, sample B and pooled samples A and B, respectively.

If homogeneity between the two samples was confirmed, then the classification was considered validated, and OR (95% CI) were estimated by conditional logistic regression on the entire sample (samples A and B combined).

## Results

### Test of the shared epitope allele classification in the new independent sample

We first observed significant over-transmission of S_2 _alleles (53 S_2_alleles transmitted versus 33.5 alleles expected; *P *= 1.9 × 10^-6^) and of S_3P _alleles (47 S_3P _alleles transmitted versus 33.5; *P *= 0.001), as was previously reported [6]. S_1_, S_3D _and X alleles were under-transmitted: 28 S_1 _alleles were transmitted versus 40 expected (*P *= 0.007), 11 S_3D _alleles was transmitted versus 18 expected (*P *= 0.02), and 30 X alleles were transmitted versus 44 expected (*P *= 0.003). These three low-risk alleles (S_1_, S_3D _and X) were pooled as L alleles, as reported previously. Thus, in subsequent analyses we considered only three alleles (S_2_, S_3P _and L alleles), with six corresponding genotypes.

The conditional logistic regression analysis provided the following hierarchy of genotype risks: S_2_/S_3P _and S_2_/S_2 _genotypes were associated with greatest risk for RA, with ORs of 19.5 and 18.0, respectively; these were followed by S_3P_/S_3P_, S_2_/L and S_3P_/L genotypes, with ORs of 8.7, 5.3 and 3.1, respectively (with the reference genotype being L/L; Table 2). This hierarchy was precisely the same as observed previously [6].

### Results of the homogeneity test

The homogeneity test on genotypic ORs between the new sample and the initial one resulted in a χ^2 ^with five degrees of freedom of 1.3 (*P *= 0.80). Because this test was not statistically significant, we considered the two samples to be homogeneous and the new classification to be valid.

### Odds ratio estimation on the pooled sample of 200 trio families

Because the two samples were homogeneous, ORs were estimated, by conditional logistic regression, for the pooled sample of 200 trio families (Table 3).

## Discussion

In the present study we validated the classification of HLA-DRB1 SE alleles in RA proposed by Tezenas du Montcel and coworkers [6]. This is the first study to validate a model of the HLA-DRB1 component of RA based on the SE hypothesis [1], with detailed investigation of the SE through the contribution of SE single amino acids to RA susceptibility, taking into account both linkage and association data. This work results in a risk genotype hierarchy, for which we provide OR estimates. The ORs were obtained exclusively from trio families, providing unbiased estimates for the sample investigated; this contrasts with estimations derived from case-control studies, for which the population matching between cases and controls can be questioned.

Further studies in other Caucasian and non-Caucasian populations are required to validate this new classification fully and investigate population-specific effects. The ORs reported here relate to relatively early onset RA, as is found in trio families. Because the mean duration of RA in both samples was long (18 years in sample A and 16 years in sample B), selection (survivor) bias would be possible even if we had considered those RA index patients who died between the time of DNA collection and the present study. Investigation of a population with common, sporadic RA is needed to assess the potential clinical relevance of this new classification. Studies with larger sample size would be able to refine the 95% CI of the OR. In the present study non-overlapping 95% CIs were observed only between the S_2_/S_3P _highest risk genotype (OR 22.2, 95% CI 9.9–49.7) and the S_3P_/L lowest risk genotype (OR 4.4, 95% CI 2.3–8.4). A significant difference between other associated genotypes remains to be established. This would provide major clues that may help in deciphering the genetic component of RA, if significant differences could be correlated with distinct pathophysiological mechanisms. It was recently reported that the SE-RA association was confined to rheumatoid factor positive patients [13] or to anti-citrullin positive RA patients [14]. The precise relationship between the HLA risk genotypes and rheumatoid factor or anti-citrullinated peptide antibodies should therefore also be determined. The interaction between HLA-DRB1 genotypes and any new RA gene established by association and linkage, such as PTPN22 [15,16], could be investigated taking this new classification into account. Ultimately, this could help in identifying other RA genetic factors that may specifically interact with only one of the HLA-DRB1 genotypes. Several previous studies indicated that other genes within HLA, such as the HLA class III region, probably contribute to RA risk [17,18]. The search for interactions between additional HLA class III genetic variants, not considered in the present study, and HLA-DRB1 genotypes taking this new classification into account would be of great interest.

Large sample size studies could refine the classification for infrequent alleles. In the present study we were unable to examine rigorously the amino acid at position 71 or at position 70, particularly for the S_1 _allele group, in which small sample size prevented study of the role played by the different alleles encoding the D-E-RAA motif. This D-E-RAA motif has been reported to be protective in the literature and constitutes an alternative SE hypothesis, although we obtained no support for it during our initial study [19]. The different S_2 _sequences Q-K-RAA (*0401) and D-K-RAA (*1303) should be evaluated separately, because the presence of an aspartic acid at position 70 has been reported to influence susceptibility to RA [20]. Similarly, the S_3P _sequences Q-R-RAA (*0101, *0102, *0404, *0405, *0408) and R-R-RAA (*1001) should be differentiated. The investigation of other amino acid positions from the third hypervariable region of the HLA-DR β-chain would be interesting, especially for positions 67 (for which the presence of an isoleucine might be important [21]) and 86 (as proposed by Gao and coworkers [22]).

Because numerous association studies have suggested that the primary role played by the SE might lie in the development of severe RA [23], the relevance of this classification should be evaluated for RA prognosis in prospective cohorts. A first investigation with the new classification already provides some support for a correlation with progression of radiographic damage [24]. Indeed, it would be of great help to be able to identify those RA patients at risk for development of more severe disease, who may require more aggressive therapeutic management than patients with better prognosis.

## Conclusion

In the present study we validated a first model of the effect of HLA-DRB1 on RA, reshaping the SE hypothesis and providing initial estimates for the resulting risk genotypes. Building on this new HLA genotype classification could lead to improvement in our understanding of the genetics, pathophysiology and potential clinical use in management of RA.

## Abbreviations

CI = confidence interval; OR = odds ratio; RA = rheumatoid arthritis; SE = shared epitope.

## Competing interests

The authors declare that they have no competing interests.

## Authors' contributions

LM, PC, EP-T, STM, FC-D and FC designed the study. LM, EP-T, IL, CP, JO, WF, SL, PQ, TB and FC acquired the data. LM, PC, EP-T, STM, BP, FCD and FC analyzed and interpreted the data. All authors read and approved the final manuscript.

## Figures and Tables

**Table 1:** Classification of HLA-DRB1 alleles

Allele classification	Amino acid sequence at positions 70–74	HLA-DRB1 alleles
S_1_	D-E-RAA	*0103, *0402, *1102, *1103, *1301, *1302, *1304, *1323
	Q-A-RAA	*15
S_2_	Q-K-RAA	*0401
	D-K-RAA	*1303
S_3D_	D-R-RAA	*1101, *1104, *12, *1305, *1306, *1325, *1422, *16
S_3P_	Q-R-RAA	*0101, *0102, *0404, *0405, *0408
	R-R-RAA	*1001
X	Q-K-RGR	*03
	Q-R-RAE	*0403, *0407, *0411
	D-R-RGQ	*07
	D-R-RAL	*08
	R-R-RAE	*0901, *1401, *1404

This classification of alleles, observed in our samples, is according to the amino acid sequence at positions 70–74, as described by Tezenas du Montcel and coworkers [6].

**Table 2:** Results of the odds ratio calculation on the replication sample (sample B)

Genotypes	Genotype distribution (*n *[%])		Odds ratio	95% Confidence interval
			
	RA index (*n *= 100)	Pseudo-controls^a ^(*n *= 300)		
S_2_/S_2_	9 (9)	11 (3.6)	18.0	4.5–71.1
S_2_/S_3P_	19 (19)	17 (5.7)	19.5	6.6–57.2
S_2_/L	23 (23)	63 (21)	5.3	2.0–13.9
S_3P_/S_3P_	5 (5)	7 (2.3)	8.7	2.1–36.9
S_3P_/L	25 (25)	77 (25.7)	3.1	1.4–6.9
L/L	19 (19)	125 (41.7)	1	-

Alleles S_2_, S_3P _and L are defined according to amino acid sequence at positions 70–74, as described in Table 1. The L alleles are the low-risk alleles S_1_, S_3D _and X. The reference genotype is L/L. ^a^Pseudo-controls are the three other genotypes that could be formed by the parental genotypes.

**Table 3:** Results of the odds ratio calculation on the global sample (samples A and B combined)

Genotypes	Genotype distribution (*n *[%])		Odds ratio	95% Confidence interval
			
	RA index (*n *= 200)	Pseudo-controls^a ^(*n *= 600)		
S_2_/S_2_	14 (7)	17 (2.8)	20.5	7.1–58.8
S_2_/S_3P_	36 (18)	34 (5.7)	22.2	9.9–49.7
S_2_/L	44 (22)	120 (20)	5.9	2.9–12.2
S_3P_/S_3P_	15 (7.5)	31 (5.2)	8.2	3.2–21.2
S_3P_/L	58 (29)	164 (27.3)	4.4	2.3–8.4
L/L	33 (16.5)	234 (39)	1	-

Alleles S_2_, S_3P _and L are defined according to amino acid sequence at positions 70–74, as described in Table 1. The L alleles are the low-risk alleles S_1_, S_3D _and X. The reference genotype is L/L. ^a^Pseudo-controls are the three other genotypes that could be formed by the parental genotypes.

## References

[B1] Gregersen PK, Silver J, Winchester RJ (1987). The shared epitope hypothesis: an approach to understanding the molecular genetics of susceptibility to rheumatoid arthritis. Arthritis Rheum.

[B2] Meyer JM, Han J, Singh R, Moxley G (1996). Sex influences on the penetrance of HLA shared-epitope genotypes for rheumatoid arthritis. Am J Hum Genet.

[B3] Génin E, Babron MC, McDermott MF, Mulcahy B, Waldron-Lynch F, Adams C, Clegg DO, Ward RH, Shanahan F, Molloy MG (1998). Modelling the major histocompatibility complex susceptibility to RA using the MASC method. Genet Epidemiol.

[B4] Rigby AS, MacGregor AJ, Thomson G (1998). HLA haplotype sharing in rheumatoid arthritis sibships: risk estimates subdivided by proband genotype. Genet Epidemiol.

[B5] Tezenas du Montcel S, Reviron D, Génin E, Roudier J, Mercier P, Clerget-Darpoux F (2000). Modeling the HLA component in rheumatoid arthritis: sensitivity to DRB1 allele frequencies. Genet Epidemiol.

[B6] Tezenas du Montcel S, Michou L, Petit-Teixeira E, Osorio J, Lemaire I, Lasbleiz S, Pierlot C, Quillet P, Bardin T, Prum B (2005). New classification of HLA-DRB1 alleles supports the shared epitope hypothesis of rheumatoid arthritis susceptibility. Arthritis Rheum.

[B7] Clerget-Darpoux F, Babron MC, Prum B, Lathrop GM, Deschamps I, Hors J (1988). A new method to test genetic models in HLA associated diseases: the MASC method. Ann Hum Genet.

[B8] Arnett FC, Edworthy SM, Bloch DA, McShane DJ, Fries JF, Cooper NS, Healey LA, Kaplan SR, Liang MH, Luthra HS (1988). The American Rheumatism Association 1987 revised criteria for the classification of rheumatoid arthritis. Arthritis Rheum.

[B9] Tsuji K, Aizawa M, Sasazuki T, editors (1992). HLA 1991. Proceedings of the 11th International Histocompatibility Workshop.

[B10] Self SG, Longton G, Kopecky KJ, Liang KY (1991). On estimating HLA/disease association with application to a study of aplastic anemia. Biometrics.

[B11] Cordell HJ, Clayton DG (2002). A unified stepwise regression procedure for evaluating the relative effects of polymorphisms within a gene using case/control or family data: application to HLA in type 1 diabetes. Am J Hum Genet.

[B12] Stata software by Clayton D. http://www-gene.cimr.cam.ac.uk/clayton/software/stata.

[B13] Padyukov L, Silva C, Stolt P, Alfredsson L, Klareskog L (2004). A gene-environment interaction between smoking and shared epitope genes in HLA-DR provides a high risk of seropositive rheumatoid arthritis. Arthritis Rheum.

[B14] Klareskog L, Stolt P, Lundberg K, Kallberg H, Bengtsson C, Grunewald J, Ronnelig J, Harris HE, Ulfgren AK, Rantapaa-Dahlqvist S (2006). A new model for an etiology of rheumatoid arthritis: smoking may trigger HLA-DR (shared epitope)-restricted immune reactions to autoantigens modified by citrullination. Arthritis Rheum.

[B15] Begovich AB, Carlton VE, Honigberg LA, Schrodi SJ, Chokkalingam AP, Alexander HC, Ardlie KG, Huang Q, Smith AM, Spoerke JM (2004). A missense single-nucleotide polymorphism in a gene encoding a protein tyrosine phosphatase (PTPN22) is associated with rheumatoid arthritis. Am J Hum Genet.

[B16] Dieudé P, Garnier S, Michou L, Petit-Teixeira E, Glikmans E, Pierlot C, Lasbleiz S, Bardin T, Prum B, Cornélis F (2005). Rheumatoid arthritis seropositive for the rheumatoid factor is linked to the protein tyrosine phosphatase nonreceptor 22–620 W allele. Arthritis Res Ther.

[B17] Jawaheer D, Li W, Graham RR, Chen W, Damle A, Xiao X, Monteiro J, Khalili H, Lee A, Lundsten R (2002). Dissecting the genetic complexity of the association between human leukocyte antigen and rheumatoid arthritis. Am J Hum Genet.

[B18] Newton JL, Harney SMJ, Wordsworth BP, Brown MA (2004). A review of the MHC genetics of rheumatoid arthritis. Genes Immun.

[B19] van der Helm-van Mil AH, Huizinga TW, Schreuder GM, Breedveld FC, de Vries RR, Toes RE (2005). An independent role of protective HLA class II alleles in rheumatoid arthritis severity and susceptibility. Arthritis Rheum.

[B20] Mattey DL, Dawes PT, Gonzalez-Gay MA, Garcia-Porrua C, Thomson W, Hajeer AH, Ollier WE (2001). HLA-DRB1 alleles encoding an aspartic acid at position 70 protect against development of rheumatoid arthritis. J Rheumatol.

[B21] de Vries N, Tijssen H, van Riel PLCM, van de Putte LBA (2002). Reshaping the shared epitope hypothesis. Arthritis Rheum.

[B22] Gao XJ, Olsen NJ, Pincus T, Stastny P (1990). HLA-DR alleles with naturally occuring amino acid substitutions and risk for development of rheumatoid arthritis. Arthritis Rheum.

[B23] Gorman JD, Criswell LA (2002). The shared epitope and severity of rheumatoid arthritis. Rheum Dis Clin North Am.

[B24] Gourraud PA, Boyer JF, Barnetche T, Abbal M, Cambon-Thomsen A, Cantagrel A, Constantin A (2006). A new classification of HLA-DRB1 alleles differentiates predisposing and protective alleles for rheumatoid arthritis structural severity. Arthritis Rheum.

